# Heat Stress Induces Apoptosis through a Ca^2+^-Mediated Mitochondrial Apoptotic Pathway in Human Umbilical Vein Endothelial Cells

**DOI:** 10.1371/journal.pone.0111083

**Published:** 2014-12-30

**Authors:** Li Li, Hongping Tan, Zhengtao Gu, Zhifeng Liu, Yan Geng, Yunsong Liu, Huasheng Tong, Youqing Tang, Junmin Qiu, Lei Su

**Affiliations:** 1 Department of intensive care unit, Guangzhou General Hospital of Guangzhou Military Command, Key Laboratory of Tropical Zone Trauma Care and Tissue Repair of PLA, Guangzhou, P.R. China, and Southern Medical University, Guangzhou, P.R. China; 2 Department of epilepsy surgery, Guangdong Sanjiu Brain Hospital, Guangzhou, P.R. China; 3 Department of intensive care unit, The Third Affiliated Hospital of Southern Medical University, Guangzhou, P.R. China; 4 Department of intensive care unit, Guangzhou General Hospital of Guangzhou Military Command, Key Laboratory of Tropical Zone Trauma Care and Tissue Repair of PLA, Guangzhou, P.R. China; University of Windsor, Canada

## Abstract

**Background:**

Heat stress can be acutely cytotoxic, and heat stress-induced apoptosis is a prominent pathological feature of heat-related illnesses, although the precise mechanisms by which heat stress triggers apoptosis are poorly defined.

**Methods:**

The percentages of viability and cell death were assessed by WST-1 and LDH release assays. Apoptosis was assayed by DNA fragmentation and caspase activity. Expression of cleaved PARP, Apaf-1, phospho-PERK, Phospho-eIF2a, ATF4, XBP-1s, ATF6, GRP78, phospho-IP3R, RYR and SERCA was estimated by Western blot. The effect of calcium overload was determined using flow cytometric analysis with the fluorescent probe Fluo-3/AM. The generation of ROS (O_2_^−^, H_2_O_2_, NO) was labeled by confocal laser scanning microscopy images of fluorescently and flow cytometry.

**Results:**

In this study, we found that heat stress in HUVEC cells activated initiators of three major unfolded protein response (UPR) signaling transduction pathways: PERK-eIF2a-ATF4, IRE1-XBP-1S and ATF6 to protect against ER stress, although activation declined over time following cessation of heat stress. Furthermore, we show that intense heat stress may induce apoptosis in HUVEC cells through the calcium-mediated mitochondrial apoptotic pathway, as indicated by elevation of cytoplasmic Ca^2+^, expression of Apaf-1, activation of caspase-9 and caspase-3, PARP cleavage, and ultimately nucleosomal DNA fragmentation; Reactive oxygen species (ROS) appear to act upstream in this process. In addition, we provide evidence that IP3R upregulation may promote influx of Ca^2+^ into the cytoplasm after heat stress.

**Conclusion:**

Our findings describe a novel mechanism for heat stress-induced apoptosis in HUVEC cells: following elevation of cytoplasm Ca^2+^, activation of the mitochondrial apoptotic pathway via the IP3R upregulation, with ROS acting as an upstream regulator of the process.

## Introduction

Environmental heat exposure can result in heat-related illnesses, and in extreme cases, can lead to death. The severity of heat-related illnesses ranges widely, from mild conditions such as heat exhaustion and heat cramps to the serious, sometimes life-threatening condition of heat stroke [1], [2]. Data from the Centers of Disease Control and Prevention indicate that from 1979 to 1997, approximately 7,000 deaths in the US were attributable to excessive heat exposure [1], [3]. In the summer of 2003, the heat wave affecting Europe resulted in an unprecedented 45,000 excessive deaths, one-third of which were due to heat stroke [4], [5]. Given the increasing intensity and frequency of heat waves as well as increasing evidence of global warming, the morbidity of heatstroke also is also likely to increase [1], [5].

Although heat-related illnesses are well-documented, the pathogenesis of cell death and tissue injury during heatstroke is poorly understood. Both *in* *vitro* and *in* *vivo* studies have demonstrated that heat stress can directly induce cell death and tissue injury [6], [7], [8]. It has been reported that exposure to extreme temperatures (49°C-50°C) compromises cellular structures and function, leading to rapid necrotic cell death in less than 5 minutes [6]. In contrast, cell death in animal models subjected to moderate heat stress proceeds by accelerated apoptosis [7]. Thus, apoptosis represents another potential mechanism of cell death in response to heat stroke. Recent molecular studies indicate a critical role for heat stress in signal transduction pathways involved in cell death; for example, induction of the apoptotic cascade through activity of apoptosis-related proteins, including caspases [9], [10]; Tissue damage by reactive oxygen species (ROS) as a result of intense heat stress is also of great concern [11], as ROS inhibit cell proliferation and activate apoptosis through induction of DNA damage [12]. Furthermore, endothelial cell apoptosis occurring early in the acute-phase response to heat stress may be a critical event in the pathogenesis of heat stroke, but the underlying mechanisms of heat stress-induced endothelial cell apoptosis are entirely unknown [13], [14].

Whether cell death is associated with elevated calcium (Ca^2+^) or ROS-dependent processes, given the highly reduced intracellular state, changes in the oxidative state are a potential trigger for cell death [15]. Elevated ROS levels cause influx of Ca^2+^ into the cytoplasm, which exacerbates oxidative stress [16]. Additionally, alterations in the redox environment of the endoplasmic reticulum (ER), which serves as the primary storage site for intracellular Ca^2+^, can result in release of Ca^2+^ from the ER through Ca^2+^-release channels [15]. Both oxidative stress and aberrantly high cytoplasmic Ca^2+^ levels can result in cytotoxicity induced by heat via activation of the apoptotic cell death program [17], [18]; however, the precise mechanisms by which heat stress induces apoptosis are poorly defined. Furthermore, mitochondria play an essential role in regulating apoptosis and cell death in response to numerous cytotoxic insults, including heat stress, via sensing oxidative stress as well as integrating and transducing the stress signal [9], [19], [20]. It has been reported that cytoplasmic Ca^2+^ overload can result in cytotoxicity, concomitant with activation of the intrinsic, or mitochondria-dependent, apoptotic pathway [21]. However, whether apoptosis of endothelial cells occurs in response to heat stress, subsequent oxidative stress, altered calcium signaling, or a combination thereof, remains to be investigated.

The objective of the present study was to explore mechanisms of heat stress-induced apoptosis in HUVEC cells. We hypothesized that heat stress-induced cytotoxicity would occur concomitant with increases in apoptotic markers, including upregulation or activation of pro-apoptotic proteins and nucleosomal DNA fragmentation. In addition to its effects on apoptosis, we also found that heat stress triggered the unfolded protein response (UPR) in order to protect cells against ER stress, although this early response declined following the cessation of heat stress. Furthermore, we demonstrate that heat stress-induced apoptosis in HUVEC cells proceeds through the calcium-mediated mitochondrial apoptotic pathway, with ROS acting upstream in this process. Finally, we show that the elevation of cytoplasmic Ca^2+^ following heat stress is mediated in part through upregulation of IP3R.

## Materials and Methods

### Cell culture, treatments and cell viability assays

Human umbilical vein endothelial cells (HUVECs) were purchased from the Shanghai Institute of Cell Biology, Chinese Academy of Sciences. Cells were grown in culture medium as recommended by the manufacturer, and used at passage 3. Cell culture dishes containing HUVEC cells were sealed with Parafilm and immersed for 2 h in a circulating water bath thermo-regulated at 37°C±0.5°C (control) or at 39°C, 41°C, 43°C, or 45°C±0.5°C (heat stress) [20], [22]. Culture medium was replaced with fresh medium and the cells were further incubated at 37°C for additional time. Cell proliferation was assessed using the Premixed WST-1 Cell Proliferation Reagent (Clontech Laboratories Inc., Mountain View, CA, USA) according to the manufacturer’s instructions. The enzymatic activity of Lactate dehydrogenase (LDH) was assayed using a commercially available kit (JianChen Co, Nanjing, China) according to the manufacturer’s instructions.

### DNA extraction and detection of DNA fragments

After exposure to 43°C heat stress for 2 h, cells were further incubated at 37°C for the indicated times. Cells were harvested by scraping and total DNA was isolated using the DNA Purification Kit (Beyotime, Beijing, China) according to the manufacturer’s protocol. DNA fragments were separated on a 0.8% agarose gel, stained with ethidium bromide, and visualized under UV light.

### Caspase activity assay

After exposure to 43°C heat stress for 2 h, cells were further incubated at 37°C for the indicated times. Cells were harvested, lysed, and cell lysates were incubated at −80°C for 30 min prior to incubation with the appropriate caspase substrates at 37°C using a Quadruple Monochromator Microplate Reader (Infinite M1000, Tecan US, NC, USA). Caspase activities were measured by cleavage of the following fluorogenic peptide substrates [23], [24]: Ac-LEHD-AFC, caspase-9; Ac-DEVD-AMC, caspase-3; Ac-ATAD-AFC, caspase-8; Ac-LEVD-AFC, caspase-4. Caspase activity is represented as relative cumulative fluorescence of the kinetic reaction relative to untreated controls.

### Plasmid construction and stable transfection

The eukaryotic Bcl-xl expression vector pcDNA3.1-Bcl-xl was constructed by Shanghai Genechem (Genechem Incorporation, Shanghai, China). HUVEC cells were transfected with either empty vector (pcDNA3.1), or pcDNA3.1-Bcl-xl using Lipofectamine 2000. Culture medium containing G418 was used to select stable transfectants.

### Ca^2+^ assay

The levels of free cytosolic calcium were measured using the cell-permeable calcium-sensitive fluorescent dye Fluo-3/AM. HUVEC cells were heat stressed at 43°C for 2 h, and further incubated at 37°C for 0, 0.5, 1, 1.5 or 2 h; HUVEC cells were incubated with 5 µM Fluo-3/AM for 30 min at 37°C. The fluorescence intensity of Fluo-3/AM probes was analyzed by flow cytometric analysis.

### Measurements of ROS

To analyze the kinetics of ROS generation,HUVEC cells were heat stressed at 43°C for 2 h, and further incubated at 37°C for 0, 0.5, 1, 1.5 or 2 h; ROS were detected using the fluorescent probe dihydroethidium (DHE, Molecular Probes), Dihydrorhodamine 123 (DHR, Molecular Probes) and 3-Amino,4-aminomethyl-2′,7′-difluorescein diacetate (DAF-FMDA, Molecular Probes). HUVEC cells were incubated with 2 µM DHE, 5 mM DHR and 5 mM DAF-FMDA, respectively for 30 min at 37°C in the dark. The fluorescence intensity of ROS probes was analyzed by flow cytometric analysis, and images were captured using laser scanning confocal microscopy.

### Western blot analysis

HUVEC cells were pretreated with or without heat stress at 43°C for 2 h, and further incubated at 37°C for different time as indicated. Western blot analysis was performed as described previously [25], [26] using the following antibodies: cleaved PARP, Apaf-1, phospho-PERK, Phospho-eIF2a, ATF4, XBP-1s, ATF6, GRP78, phospho-IP3R, RYR and SERCA (all used at 1∶1000; Cell Signaling Technology, Danvers, USA). An HRP-conjugated anti-rabbit IgG antibody was used as the secondary antibody (Zhongshan Inc, China), and signal was visualized enhanced chemiluminescence (Pierce, Rockford, IL, USA).

### RNA isolation, reverse transcription, qRT-PCR

Total RNA was extracted from HUVEC cells using Trizol (Takara, Shiga, Japan). RNA was transcribed into cDNA and amplified with specific sense: 5′-GCAGGTGCTAG-3′ and antisense primers: 5′-CAGTTATATCTCTC-3′. Reverse transcription and qPCR were performed in accordance with manufacturer’s instructions (Takara, Shiga, Japan). qPCR reactions for each gene were performed in triplicate. EDEM expression was normalized relative to the housekeeping gene GAPDH. Differential expression of EDEM was calculated using the 2−ΔΔCt method.

### Statistical analysis

All data were analyzed for statistical significance using SPSS 13.0 software (SPSS, Chicago, IL, USA). Data were expressed as mean ± SD from at least 3 independent experiments performed in duplicate. Statistical comparisons of the results were performed using One-way analysis of variance (ANOVA). A P value <0.05 was considered to be statistically significant.

## Results

### 1. Effects of heat stress on HUVEC cell viability and cytotoxicity

**Figure 1:**
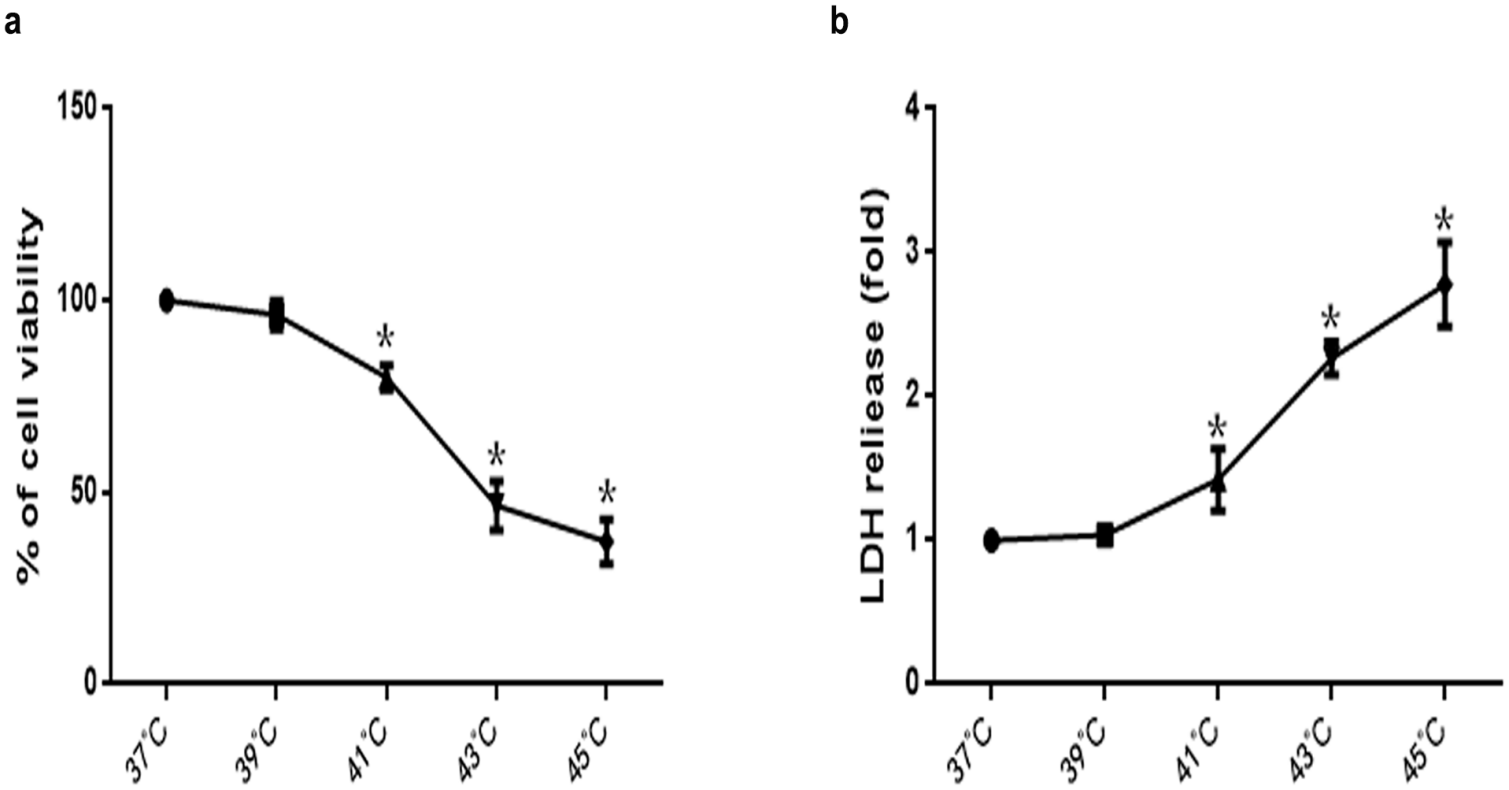
Heat stress reduces cell viability and increased cytotoxicity in HUVEC cells. Cells were exposed to the indicated temperature for 2 h, and were further incubated at 37°C for 6 h. The percentages of viability and cell death were assessed by WST-1 (a) and LDH release assays (b). Percent viability is expressed relative to control cells cultured at 37°C. The data shown represent the mean ±SD of at least three independent experiments, peformed in triplicate. *P<0.05, statistically significant relative to control.

To investigate changes in viability and cytotoxicity following heat stress in HUVEC cells exposed to elevated temperatures, both the WST-1 and LDH assays were employed. HUVEC cells were maintained in standard culture media for 48 h at 37°C prior to a temperature shift to 39°C, 41°C, 43°C, or 45°C for the duration of heat stress treatments. Culture media were replaced with fresh media and cells were further incubated at 37°C for 6 h. Cell viability declined drastically concomitant with a significant increase in cytotoxicity after cells were cultured at elevated temperatures, as indicated by the temperature-dependent reduction in formazan formation and increase in LDH activity, respectively (**Fig. 1a and b**).

### 2. Intense heat stress induces DNA fragmentation and activates caspase-3 and PARP in HUVEC cells

**Figure 2:**
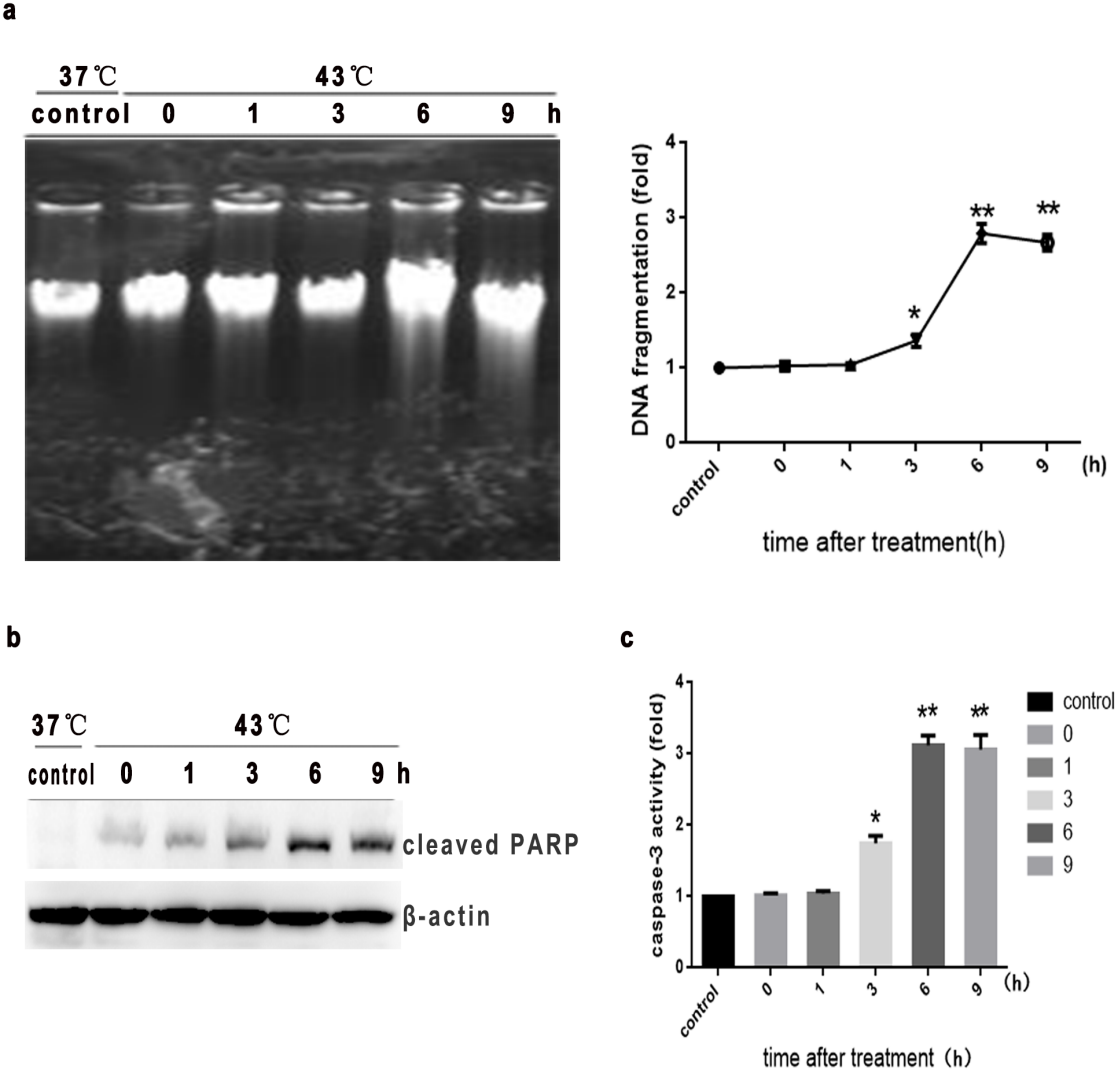
Intense heat stress induces apoptotic DNA fragmentation and activates caspase-3 and PARP in HUVEC cells. Cells underwent intense heat stress (43°C) for 2 h, and were further incubated at 37°C for different times as indicated (0 h, 1 h, 3 h, 6 h, or 9 h). (a) DNA fragmentation using DNA electrophoresis and fluorescent staining. (b) Western blot analysis of cleaved PARP. (c) Enzymatic activity of caspase-3 was measured in cell lysates using the fluorogenic substrate Ac-DEVD-AMC. Each value is the mean ± SD of at least three separate experiments, *P<0.05, **P<0.01, compared with control group (37°C).

To quantitatively assess heat stress-induced apoptosis in HUVEC cells, we first examined nucleosomal DNA fragmentation. The classical apoptotic DNA laddering pattern was observed between 3 hr and 9 h following a 2 hr period of intense heat stress (43°C), suggesting that heat stress-induced cytotoxicity occurs through activation of apoptosis (**Fig. 2a**). Since various stress signals activate the caspase cascade, we investigated enzymatic activity of caspase-3 after heat stress in HUVEC cells. Using the fluorogenic substrate Ac-DEVD-AMC, we observed caspase-3 activity at 3 h, 6 h, and 9 h after a 2 hr period of intense heat stress (43°C) (**Fig. 2c**), which was consistent with the time period during which PARP cleavage occurred (**Fig. 2b**). Thus caspase seems to the primary executioner in heat stress-induced apoptosis of HUVEC cells.

### 3. Intense heat stress induces the endoplasmic reticulum (ER) stress-related unfolded protein response (UPR) in HUVEC cells

**Figure 3:**
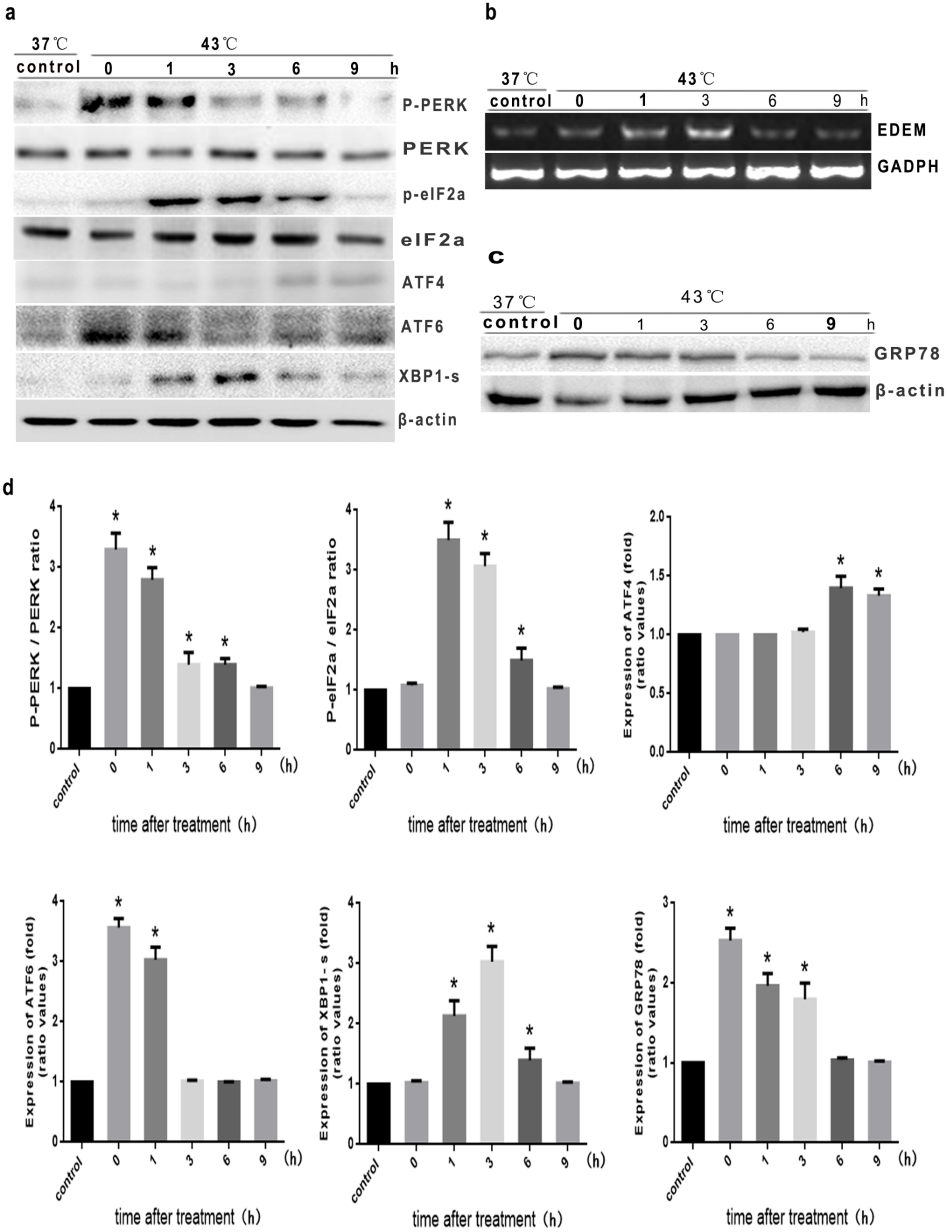
Intense heat stress induces the UPR in HUVEC cells. Cells were exposed to intense heat stress (43°C) for 2 h, and were further incubated at 37°C for 0 h, 1 h, 3 h, 6 h, or 9 h. (a) and (c) Expression of P-PERK, P-elF2a, ATF4, ATF6, XBP1-s, and GRP78 were determined by Western Blot. (b) RT-qPCR analysis of XBP-1s target gene expression (EDEM). (d) Quantification of western blots for PERK phosphorylation, eIF2a phosphorylation, ATF4, ATF6, XBP1-s and GRP78 after heat stress. Graphs represent mean±S.D. of at least three independent experiments. *P<0.05, compared with control group (37°C).

ER stress can result from alterations in the luminal ER environment; activation of the unfolded protein response (UPR) represents one mechanism by which cells respond to the induction of ER stress [15]. To explore whether intense heat stress induces ER stress and activates the UPR, we investigated the ER stress-related proteins, including phospho-PERK, phospho-eIF2a, ATF4, XBP-1s, ATF6 and GRP78, as well as the transcription of the XBP-1s target gene EDEM. As shown in **Fig. 3a, d**, PERK phosphorylation was gradually inhibited immediately following a 2 hr heat stress; eIF2a phosphorylation was inhibited in a time-dependent manner beginning 3 hr after the cessation of heat stress. ATF4 expression was induced by 6 hr after heat stress, and declined thereafter. Activation of ATF6 peaked immediately after heat stress (0 hr), then gradually decreased to baseline. Expression of XBP-1s was observed as early as 1 hr after heat stress, peaking at 3 hr, then gradually declining; the alterations in EDEM mRNA levels are consistent with the dynamic changes in XBP-1s (**Fig. 3b, d**). GRP78 was activated at 0 h and decreased gradually by 3 h after cessation of heat stress (**Fig. 3c, d**). Taken together, these results suggest that intense heat stress triggers the UPR to protect cells against ER stress, which activates three UPR transducer pathways: PERK-eIF2a-ATF4, IRE1-XBP-1S and ATF6; however, the activation of these unfolded proteins decreased at later time points.

### 4. Intense heat stress induces apoptosis by triggering the mitochondrial pathway in HUVEC cells

**Figure 4:**
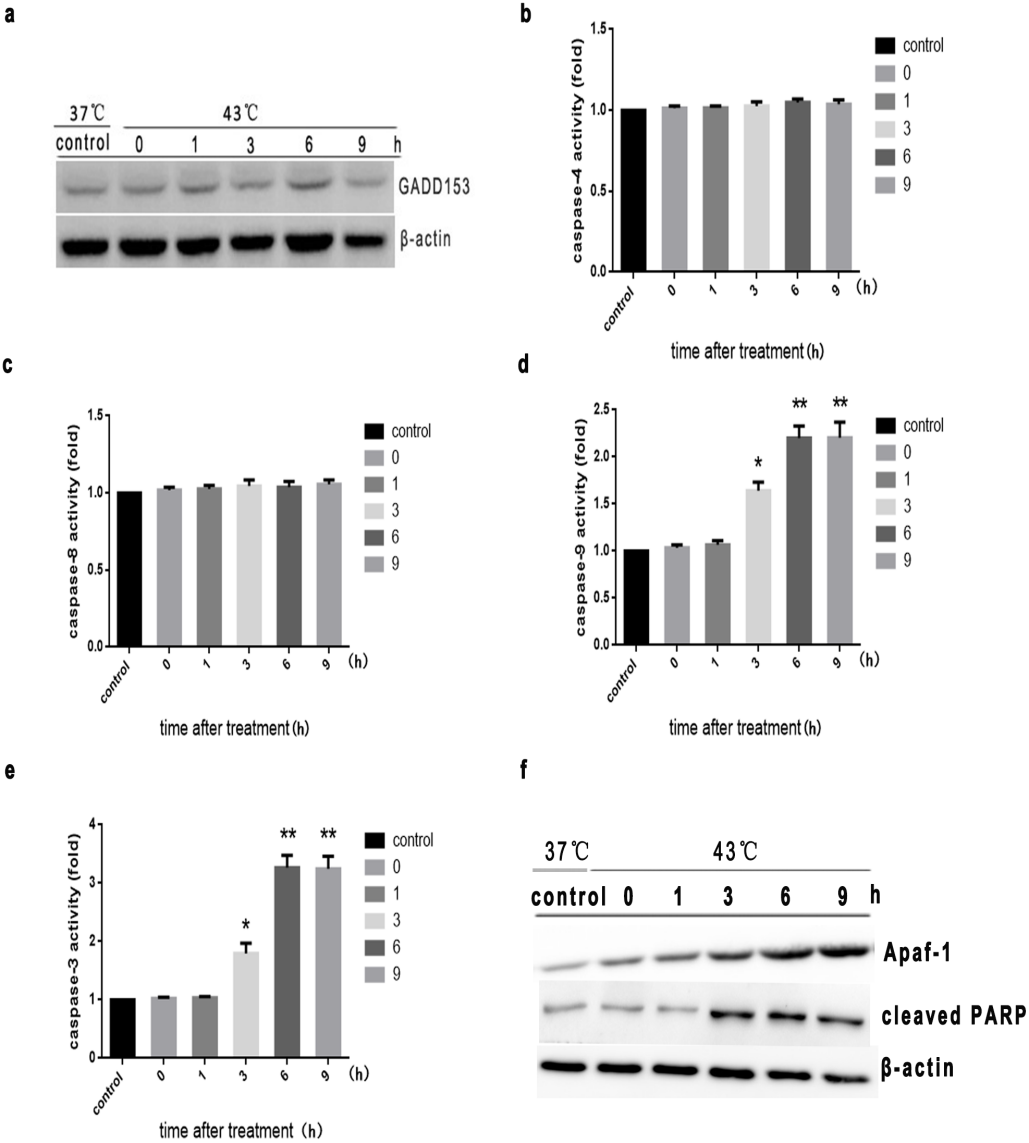
Intense heat stress induces apoptosis by triggering the mitochondrial pathway in HUVEC cells. Cells udnerwent intense heat stress (43°C) for 2 h, and were further incubated at 37°C for the indicated times (0 h, 1 h, 3 h, 6 h, or 9 h). (a) and (f) Expression of GADD153, Apaf-1 and cleaved PARP were determined by Western blot. (b–e) Enzymatic activity of caspase-9, -3, -8 and -4 were measured in cell lysates using the fluorogenic substrates Ac-LEHD-AFC, Ac-DEVD-AMC, Ac-ATAD-AFC and Ac-IETD-pNA, respectively, and was expressed relative to the control at 37°C. Data are shown as the mean ± SD of at least three independent experiments, *P<0.05, **P<0.01, compared with control group (37°C).

Based on our findings that intense heat stress induced ER stress-related UPR signal transduction pathways, we next investigated whether intense heat stress also activates the apoptotic ER stress response pathway. First, we examined expression of GADD153, a critical mediator of ER stress-induced apoptosis [27]. As shown in **Fig. 4a** and **Fig. 4b**, there was no apparent increase in GADD153 expression or caspase-4 activity in cells undergoing heat stress, suggesting that intense heat stress did not induce the apoptotic ER stress response pathway in HUVEC cells.

**Figure 5:**
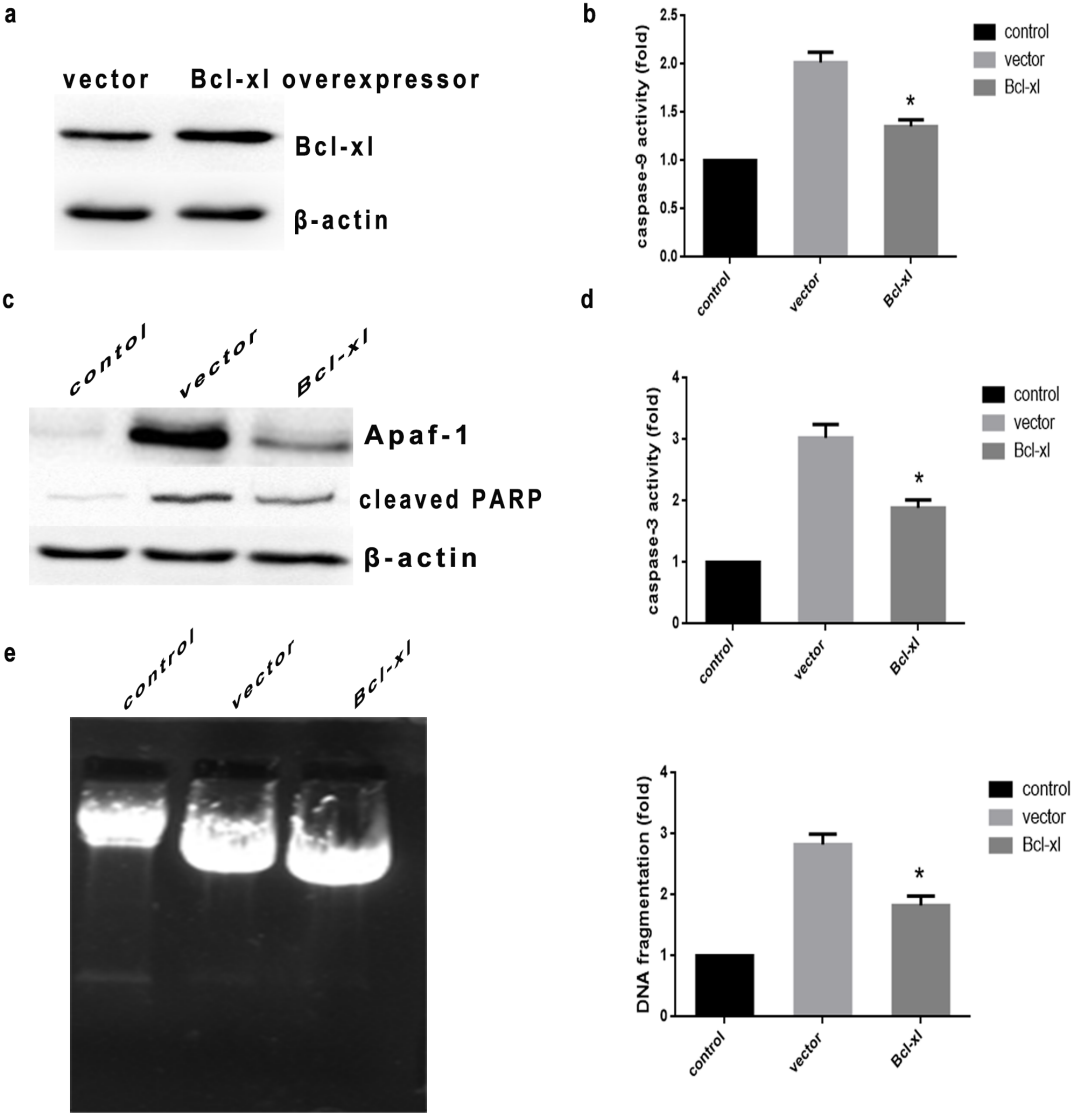
Intense heat stress-induced apoptosis in HUVEC cells over-expressing Bcl-xl. HUVEC cells stably overexpressing the anti-apoptotic protein Bcl-xl or vector control were cultured at 43°C for 2 h, and further incubated for 6 h. (a) Western blot analysis of bcl-2 protein expression (cropped) in transfected cells. β-actin served as an internal control. (b) and (d) Enzymatic activity of caspase-9, and -3 was measured in cell lysates using the fluorogenic substrates Ac-LEHD-AFC and Ac-DEVD-AMC, respectively, and was expressed relative to the control at 37°C. (c) Expression of Apaf-1 and cleaved PARP were determined by Western blot. (e) Nucleosomal DNA fragmentation was evaluated using DNA electrophoresis and fluorescent staining. Each value is the mean ± SD of at least three separate experiments, *P<0.05, compared with heat stress group (43°C).

In order to identify the mechanistic pathways involved in heat stress-induced apoptosis, we examined alterations in the activity of initiator caspases (caspase-8, -9) and the main effector caspase (caspase-3). As shown in **Fig. 4c**, caspase-8 was not activated, whereas activation of caspase-9 and caspase-3 was detected 3 h after heat stress. Caspase-9 and -3 activity increased by more than 2-fold and 3-fold, respectively (**Fig. 4d, e**). Also, Apaf-1 expression was elevated and PARP cleavage was observed 3 h after heat stress, consistent with increased caspase-9 and -3 activity (**Fig. 4f**), suggesting that heat stress might induce the mitochondrial apoptotic pathway. As shown in **Fig. 5**, Bcl-xl overexpression significantly decreased heat stress-induced apoptosis, further supporting the hypothesis that heat stress-induced apoptosis of HUVEC cells was mediated by the mitochondrial pathway.

### 5. Calcium elevation induced by intense heat stress activates mitochondrial apoptosis pathways in HUVEC cells

**Figure 6:**
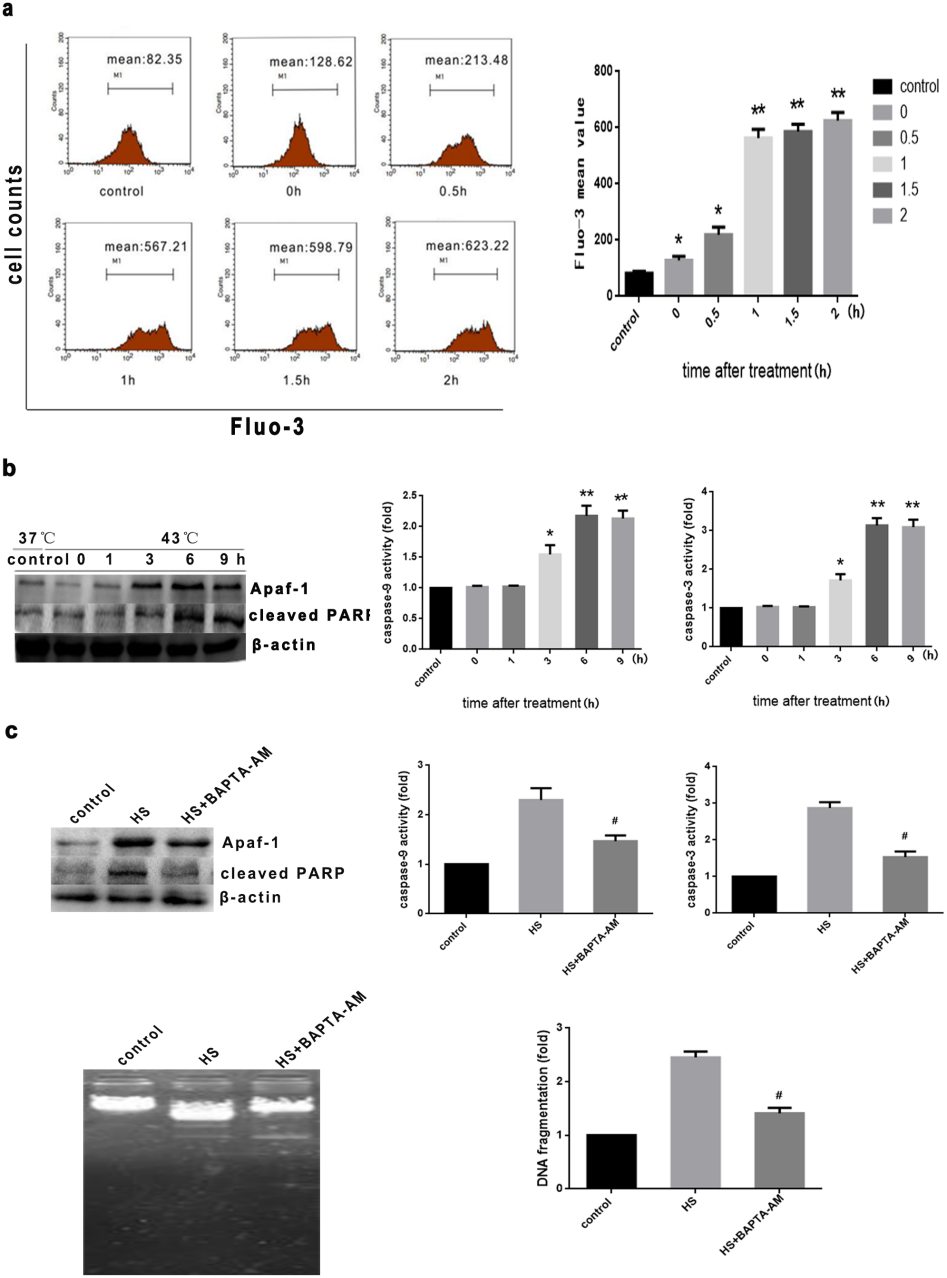
Calcium elevation induced by intense heat stress activates the mitochondrial apoptotic pathway. (a) Cells underwent intense heat stress (43°C) for 2 h, and were further incubated at 37°C for the indicated times (0 h, 0.5 h, 1 h, 1.5 h, 2 h). The effect of heat stress on calcium overload was determined using flow cytometric analysis with the fluorescent probe Fluo-3/AM. (b) Cells underwent heat stress as described above, and were further incubated at 37°C for the indicated times (0 h, 1 h, 3 h, 6 h, or 9 h). (c) Cells were pretreated with 20 M BAPTA-AM, then exposed to intense heat stress (43°C) for 2 h, and incubated at 37°C for 6 h. Expression of Apaf-1 and cleaved PARP were determined by Western blot analysis. Enzymatic activity of caspase-9 and -3 was measured in cell lysates using the fluorogenic substrates Ac-LEHD-AFC and Ac-DEVD-AMC, respectively, and activity was expressed relative to the control at 37°C. DNA fragmentation using DNA electrophoresis and fluorescent staining. Each value represents the mean ± SD of at least three separate experiments, *P<0.05, **P<0.01, compared to control group (37°C),^ #^ *P*<0.05, compared to heat stress group (43°C).

Calcium (Ca^2+^) is a critical second messenger involved in many cellular processes including fertilization, proliferation, differentiation, secretion, gene expression, muscle contraction and apoptosis. Several studies have demonstrated increased [Ca^2+^] during both early and late stages of apoptosis [28], [29]. To investigate whether heat stress-induced apoptosis is associated with increased intracellular calcium, we determined the effect of Ca^2+^ on heat stress-induced apoptosis using flow cytometric analysis of cells were stained with the fluorescent probe Fluo-3/AM. Heat stress caused an increase in the level of intracellular Ca^2+^ within 2 hr, which further increased 2 hr after the cessation of heat stress, at which time the intracellular calcium concentration was significantly higher than control (*P*<0.05 or <0.01, **Fig. 6a**). To determine whether increased intracellular calcium induced by heat stress is involved in regulating the activation of proapoptotic proteins through the intrinsic pathway, we measured enzymatic activity of caspase-9, caspase-3 and expression of Apaf-1 and cleaved PARP. As shown in **Fig. 6b**, the activities of caspase-9, caspase-3 and the expression of Apaf-1 and cleaved PARP were clearly elevated 3 h after heat stress and continued to increase up to 9 h. Pretreatment with the cell-permeant calcium chelator BAPTA-AM significantly decreased apoptosis 6 h after intense heat stress (**Fig. 6c**). Taken together, these results indicate that intense heat stress induced a time-dependent increase in intracellular calcium elevation, which regulated activation of proapoptotic mediators through the mitochondrial-mediated apoptosis pathway.

### 6. Effect of intense heat stress on the expression levels of Ca^2+^ channel release related protein (IP3R)

**Figure 7:**
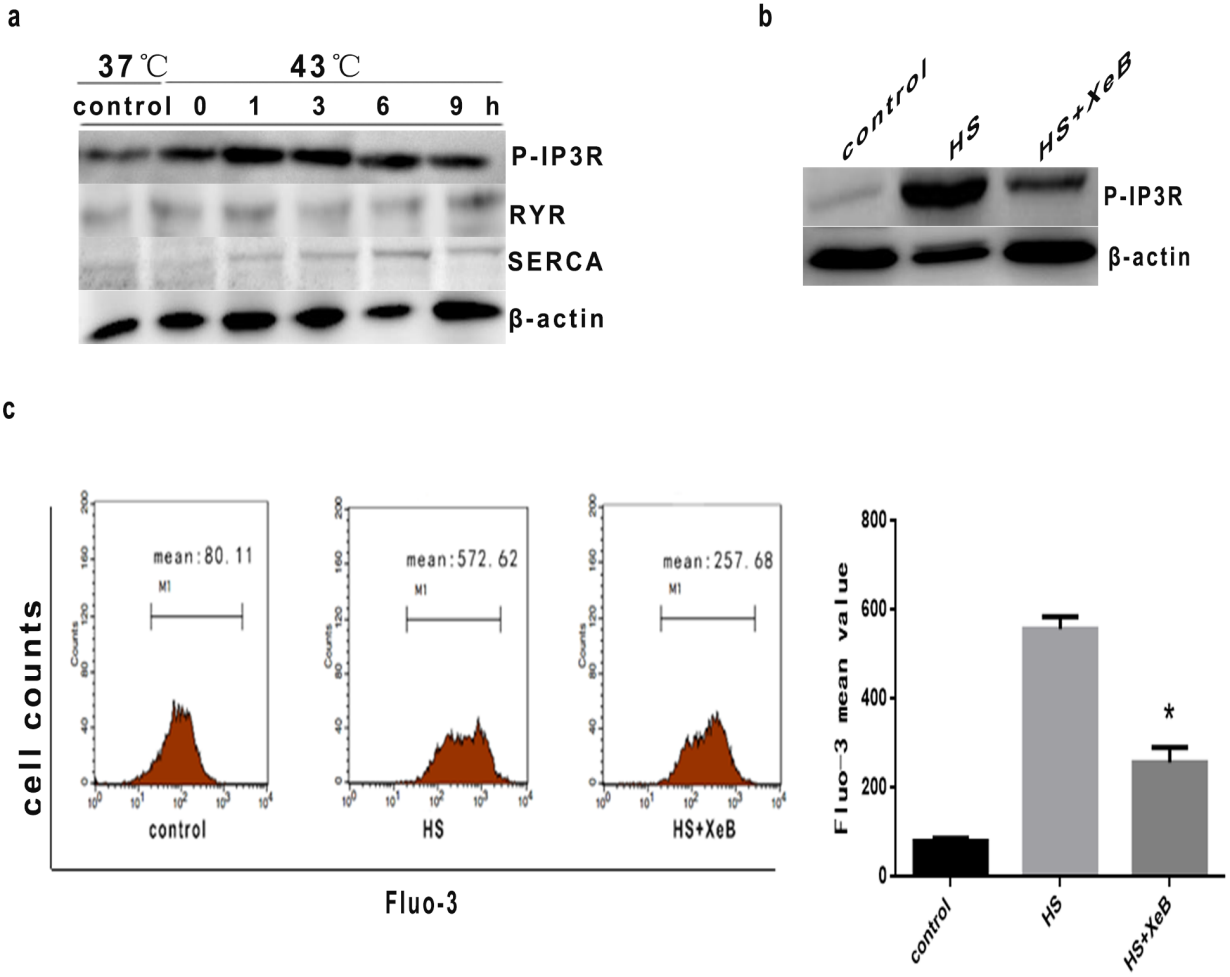
Effect of intense heat stress on the expression of Ca^2+^channel release related protein (IP3R). (a) Cells were exposed to intense heat (43°C) for 2 h, and were further incubated at 37°C for 0 h, 1 h, 3 h, 6 h, or 9 h. Expression of phospho-IP3R, RYR, and SERCA were determined by Western blot. (b) and (c) Cells were treated with 2.5 µM Xestospongin B(XeB) prior to heat stress, and further incubated at 37°C for 1 h; phosphorylation of IP3R was determined by Western blot analysis. The effect of heat stress on calcium overload was determined by flow cytometric analysis of cells labeled with the fluorescent probe Fluo-3/AM. Each value represents the mean ± SD of at least three independent experiments; *P<0.05, compared to the heat stress group (43°C).

Two major families of Ca^2+^ release channels have been identified, the inositol 1,4,5-triphosphate receptors (IP3Rs) [30] and the ryanodine receptors (RyRs) [31], while the sarco-endoplasmic reticulum Ca^2+^ ATPase (SERCA) serves to pump cytosolic calcium back into internal stores [32]. To investigate which Ca^2+^ channels were relevant to ER stress induced by high temperatures, we evaluated expression of IP3R, RYR, and SERCA in HUVEC cells. As shown in **Fig. 7a**, IP3R phosphorylation was observed immediately following heat stress (0 h), and increased significantly up to 9 h. Since no obvious increase in RYR or SERCA expression was observed following heat stress, we concluded that IP3R is primarily responsible for mediating the regulatory effects of heat stress on Ca^2+^ release. It has been reported that the effects of increased cytoplasmic Ca^2+^ can be counteracted using the selective IP3R antagonist xestospongin B (XeB) [33]. Pretreatment of HUVEC cells with 2.5 µM XeB reduced the IP3R phosphorylation within 1 hr of cessation of intense heat stress (**Fig. 7b**); XeB also reduced the release of Ca^2+^ by approximately 45% (**Fig. 7c**), consistent with IP3R mediating the effects of heat stress on Ca^2+^ release.

### 7. ROS involved in the elevation of calcium-mediated mitochondrial apoptotic pathway induced by intense heat stress in HUVEC cells

**Figure 8:**
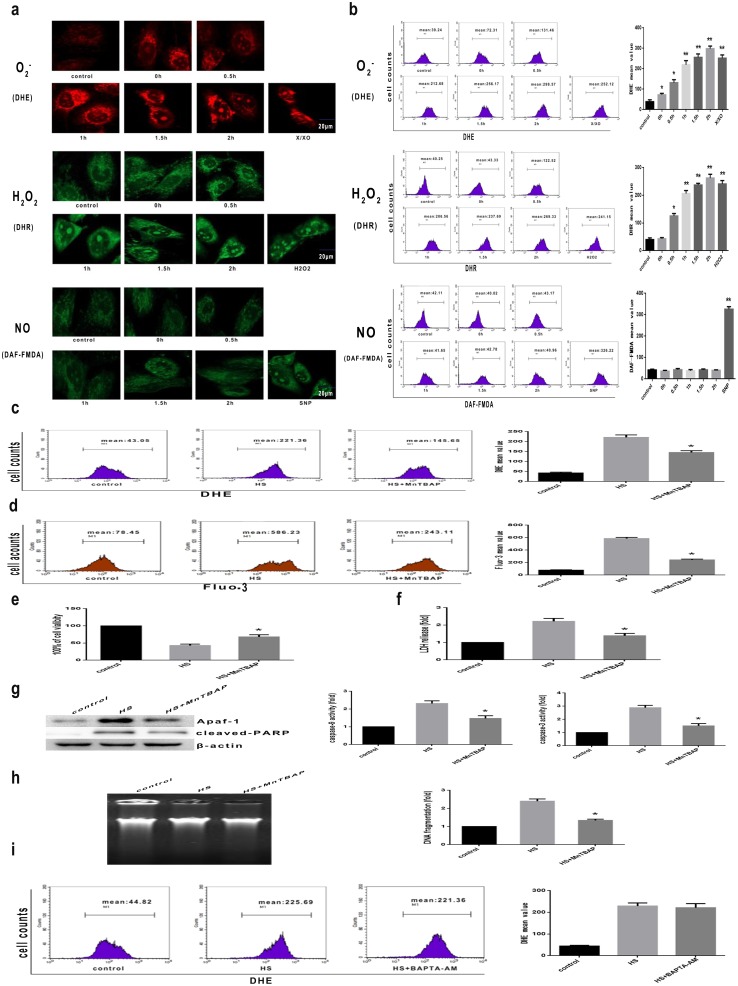
ROS is involved in induction of the calcium-mediated mitochondrial apoptotic pathway induced by intense heat stress in HUVEC cells. (a)–(b) Cells were exposed to intense heat (43°C) for 2 h, then incubated at 37°C for 0 h, 0.5 h, 1 h, 1.5 h, or 2 h. Cells were labeled with DHE (red fluorescence), DHR (green fluorescence), or DAF-FMDA (green fluorescence) for detection of superoxide, H_2_O_2_, or nitric oxide, respectively. X/XO (0.05 mM X+0.01 U XO), H_2_O_2_ (25 µM) and SNP (0.01 mol/L) were used as a positive control for O_2_^−^, H_2_O_2_ and NO, respectively. (a) Confocal laser scanning microscopy images of fluorescently labeled cells. Representative images of the best of three independent image acquisitions are shown. (b) Flow cytometry analysis heat stress-induced ROS (O_2_^−^, H_2_O_2_, NO). (c)–(d) Cells were pretreated with MnTBAP prior to 2 hr of heat stress (43°C), then further incubated at 37°C for 1 h. (c) Flow cytometric analysis of ROS generation (O_2_^−^). (d) The effect of heat stress on calcium overload was analyzed by flow cytometry using the fluorescent probe Fluo-3/AM. (e)–(h) Cells were pretreated with MnTBAP prior to heat stress (43°C) for 2 h, and further incubated at 37°C for 6 h. Cell death was assessed by the WST-1 (e) and LDH release assays (f). (g) Expression of Apaf-1 and cleaved PARP were determined by Western blot analysis. Enzymatic activity of caspase-9 and -3 was measured in cell lysates using the fluorogenic substrates Ac-LEHD-AFC and Ac-DEVD-AMC, respectively; caspase activity was expressed relative to the control at 37°C. (h) Nucleosomal DNA fragmentation using DNA electrophoresis and fluorescent staining. (i) Cells were pretreated with 20 M BAPTA-AM, exposed to intense heat (43°C) for 2 h, and incubated at 37°C for 1 h. Flow cytometric analysis of ROS (O_2_^−^) was performed as in (c). Each value represents the mean ± SD of at least three independent experiments; *P<0.05, compared with heat stress group (43°C).

Given that ROS generation plays an important role in the cellular response to heat stress [17], we hypothesized that heat stress-induced apoptosis potentiates the accumulation of ROS. To test this hypothesis, we monitored intracellular ROS production using the cell-permeant fluorescent dyes DHE, DHR and DAF-FMDA, whose fluorescence is enhanced under ROS (O_2_^−^, H_2_O_2,_ NO) generating conditions. X/XO, H_2_O_2_ and SNP -treated HUVEC cells were used as positive controls. Heat stress induced production of both O_2_^−^ and H_2_O_2_ reactive species_;_ however, O_2_^−^ noticeably increased immediately after heat stress (0 h), while H_2_O_2_ increased significantly 0.5 h after heat stress; both free radical species continued to increase with time thereafter (**Fig. 8a**). As shown in **Fig. 8b**, DHE and DHR exhibited similar increases in fluorescence intensity after heat stress. Thus, heat stress induced time-dependent increases in both O_2_^−^ and H_2_O_2,_ with the increase in O_2_^−^ preceding the increase in H_2_O_2_; in contrast, NO generation did not change significantly.

ROS have the potential to damage cellular components, and elevation of calcium levels is closely related to ROS generation [16]. The above results indicated that heat stress first induced an increase in O_2_^−^ free radical followed by increased intracellular calcium levels. To explore the relationship between ROS generation and calcium levels, we pretreated cells with the O_2_^−^ scavenger MnTBAP and the calcium chelator BAPTA-AM. As shown in **Fig. 8c, d**, increases in both O_2_^−^ and cytoplasmic Ca^2+^ were completely inhibited by MnTBAP. MnTBAP pretreatment increased viability and reduced cytotoxicity following heat stress in HUVEC cells (**Fig. 8e, f**). The results in **Fig. 8g, h**, confirm that while MnTBA also significantly decreased heat stress-induced apoptosis mediated by the mitochondrial pathway, O_2_^−^ production was not inhibited by BAPTA-AM (**Fig. 8i**). Overall, these results indicate that ROS generated by intense heat stress acts as the upstream stimulus for elevation of calcium levels as well as downstream activation of the mitochondrial apoptotic pathway in HUVEC cells.

## Discussion

Heat is the most fundamental factor in the pathogenesis of heat stroke and can be directly toxic to cells [13]. Temperature elevation can result in vascular endothelium injury, and it has been reported that the endothelial cell is the primary cell population affected during severe heat stroke. The endothelial cell also is an early target in heat stress injury [14], [34], thus the mechanisms of endothelial cell injury and cell death are highly relevant to understanding the pathogenesis of heat stroke. Our earlier clinical trials found that patients with severe heat stroke present with serious vascular endothelial cell injury, and previous studies in HUVEC cells found that inhibition of endothelial cell proliferation directly contributed to the cytotoxic effects of heat stress [20]. Our previous work also confirmed that endothelial cell apoptosis may be mechanistically relevant to the pathogenesis of heat stroke [20]. Here, we expand our investigation to the temperature- and time-dependent effects of heat stress on endothelial cell apoptosis, including the relevant signaling pathways, upstream signaling molecules and cross-talk between signaling intermediates.

The endoplasmic reticulum (ER) is a specialized organelle involved in numerous cellular functions, including the synthesis, folding, and maturation of proteins, as well as in intracellular calcium storage and release [35]. ER stress provoked by an imbalance between the capacity and the load of unfolded proteins in the ER results in the accumulation of unfolded or misfolded proteins in the ER lumen [36]. Cells are protected against ER stress through activation of the UPR [37]; however, under conditions which induce chronic or severe ER stress, the UPR fails to promote cell survival, and ER stress ultimately leads to activation of apoptosis [37], [38]. The UPR signaling pathway is mediated by three major ER stress sensors, PKR-like ER kinase (PERK), inositol requiring enzyme 1 (IRE1), activating transcription factor 6 (ATF6), and an ER resident chaperone, glucose-regulated protein 78 (GRP78) [15], [37]. Our studies indicate that intense heat stress activates the initiators of these UPR signal transduction pathways: PERK-eIF2a-ATF4, IRE1-XBP-1S and ATF6. Heat stress first induces phosphorylation and activation of PERK, which phosphorylates eIF2a, a translation initiation factor which can attenuate protein synthesis and protect cells against ER stress; eIF2a selectively induces translation of ATF-4, which potentiates transcriptional activation of the proapoptotic factor GADD153[39]. Results shown here indicate that elevated ATF4 expression occurred within 6 hr following heat stress, but that its expression decreased with time. The IRE-1 protein can dimerize and autoactivate its endoribonuclease activity; active IRE-1 cleaves XBP-1 mRNA, converting unspliced XBP-1u to its spliced form XBP-1s [15]. Formation of XBP-1s can result in activation of additional, more potent transcriptional activators, including the protein degradation gene EDEM. Our results indicate that XBP-1 activation was induced within 1 h following heat stress, then was gradually inhibited, consistent with the observed trends in XBP-1 expression. Thus, IRE-1 induced the transcription of EDEM via XBP-1 to reduce protein accumulation and protect cells against ER stress after heat stress. ATF6 is one of the key factors mediated cell survival and exerts primarily adaptive functions during ER stress [40]. ATF6 expression and cleavage are induced early after heat stress, after which ATF6 translocates to the nucleus to induce expression of GRP78 [15], [41].

In this study, we found that heat stress increased nucleosomal DNA fragmentation, caspase-3 activity and PARP cleavage in a time-dependent manner, indicating that heat stress can activate the apoptotic program. We have also shown that UPR signal transduction pathways were activated early after heat stress, although their activation declined over time after cessation of heat stress. In other words, the UPR failed to promote cell survival and the ER-dependent survival pathway was inhibited at later stages following heat stress. To determine whether intense heat stress induces apoptosis through activation of the ER stress response pathway, we evaluated changes in GADD153 expression and caspase-4 activity, both of which are the critical determinants of apoptosis mediated by ER stress [27]. However, given the absence of a noticeable increase in GADD153 or caspase-4 activity, we concluded that intense heat stress did not induce the apoptotic ER stress response pathway in HUVEC cells.

Calcium is one of the most crucial intracellular messengers, which functions to translate extracellular stimuli into intracellular signaling pathways that ultimately regulate cellular development, survival, differentiation and gene expression [41]. Intracellular calcium concentration must be precisely regulated, and this delicate balance is maintained by extracellular membrane calcium channels, intracellular calcium release channels and calcium pumps, and exchangers [41], [42], [43]. ER stress can affect Ca^2+^ release, and accumulation of unfolded proteins in the ER can result in Ca^2+^ leakage into the cytosol [15]. Ca^2+^ release from internal stores is largely mediated by two intracellular Ca^2+^-release channels: IP3Rs and RyRs [30], [31], which are widely thought to be vital modulators of Ca^2+^ release [44], [45]. Our research indicates that IP3R was phosphorylated immediately after heat stress, and preceded a time-dependent increase in cytosolic Ca^2+^ levels. While there was no obvious increase in RYR or SERCA in heat-stressed cells, further studies confirmed that the selective IP3R antagonist XeB reduced Ca^2+^ release by nearly 45%. Therefore, we posit that the regulatory effects of heat stress on the release of Ca^2+^ are mediated by upregulation of IP3R.

Increased cytosolic Ca^2+^ concentration is a potent activator of the intrinsic apoptotic pathway. Disruption of Ca^2+^ homeostasis induces a series of biochemical alterations leading to caspase activation and subsequent cellular apoptosis [46], [47]. Mitochondria are the central integrators and transducers of proapoptotic signals, and Hsu et al. found that heat stress triggered the mitochondrial apoptotic pathway, resulting in caspase-9 activity [9]. In the present study, we detected increases in caspase-9 and -3 activity, Apaf-1 expression, PARP cleavage, and nucleosomal DNA fragmentation, but not caspase-4 or -8 activity, after heat stress. This is consistent with intense heat stress initiating the mitochondrial apoptotic pathway in HUVEC cells independent of the ER or death receptors. Pretreatment of cells with the calcium chelator BAPTA-AM significantly decreased heat stress-induced mitochondrial-mediated apoptosis, revealing that the increased intracellular calcium as a result of heat stress is involved in regulating release of proapoptotic proteins through the mitochondrial pathway.

While basal levels of ROS contribute to normal cellular functions and intracellular signaling, increased ROS levels through exposure to cytotoxic agents, including irradiation or environmental pollutants, or during enzymatic reactions can induce oxidative stress and cell death [15]. It has been reported previously that heat stress and heat-induced ROS generation may act in concert to promote cell death [17], [48], [49]. In this study, we discovered that heat stress increases formation of two types of oxygen free radicals in a time-dependent manner: O_2_^−^ and H_2_O_2,_ with the change in O_2_^−^ occurring early and potentiating an increase in H_2_O_2_; in contrast, NO levels were unaffected by heat stress. Therefore, excessive generation of O_2_^−^ free radicals may be the primary mechanism of ROS generation after heat stress. Furthermore, recent studies have proposed that ROS acts both as an upstream stimulus triggering intracellular signal transduction cascades during Ca^2+^-mediated apoptosis, and as a downstream factor mediating apoptosis [50]. Pretreatment of cells with the O_2_^−^ scavenger MnTBAP demonstrated that ROS generated by intense heat stress, particularly the O_2_^−^ free radical, acts as an upstream stimulus involved in the elevation of cytosolic calcium levels and activation of the mitochondrial apoptotic pathway in HUVEC cells.

In conclusion, our present work indicates that intense heat stress triggers the UPR to protect cells against ER stress, whereas the activation of UPR declines with time after the cessation of heat stress. Heat stress might initiate mitochondrial signaling pathways independent of the ER or death receptors to promote apoptosis through the elevation of intracellular calcium levels. In addition, the elevation in cytoplasmic Ca^2+^ was in part mediated by IP3R upregulation after heat stress. ROS, particularly O_2_^−^, acts as an upstream signaling molecule involved in heat stress-induced HUVEC cell apoptosis. However, it remains to be studied, whether such regulatory mechanisms occur similar in vivo and in other cell systems.

## Supporting Information

S1 Data (ZIP)
